# Quirky conversations: how people with a diagnosis of schizophrenia do dialogue differently

**DOI:** 10.1098/rstb.2021.0480

**Published:** 2023-04-24

**Authors:** Christine Howes, Mary Lavelle

**Affiliations:** ^1^ Department of Philosophy, Linguistics and Theory of Science, University of Gothenburg, 405 30 Gothenburg, Sweden; ^2^ School of Psychology, Queens University, Belfast BT7 1NN, UK

**Keywords:** dialogue, schizophrenia, turn-taking, gesture, repair

## Abstract

People with a diagnosis of schizophrenia (PSz) have difficulty engaging in social interaction, but little research has focused on dialogues involving PSz interacting with partners who are unaware of their diagnosis. Using quantitative and qualitative methods on a unique corpus of triadic dialogues of PSz first social encounters, we show that turn-taking is disrupted in dialogues involving a PSz. Specifically, there are on average longer gaps between turns in groups which contain a PSz compared to those which do not, particularly when the speaker switch occurs from one control (C) participant to the other. Furthermore, the expected link between gesture and repair is not present in dialogues with a PSz, particularly for C participants interacting with a PSz. As well as offering some insights into how the presence of a PSz affects an interaction, our results also demonstrate the flexibility of our mechanisms for interaction.

This article is part of a discussion meeting issue ‘Face2face: advancing the science of social interaction’.

## 1. Introduction

Schizophrenia is diagnosed in approximately 1% of the population and is characterized by social dysfunction (DSM-IV). Difficulty engaging in social interaction is one of the most debilitating aspects of the disorder with significant consequences for the lives of those diagnosed. People with a diagnosis of schizophrenia (PSz) have low rates of employment [1], smaller social networks, and are one of the most socially excluded groups in society [2]. Social functioning difficulties are present prior to the onset of other diagnostic symptoms such as hallucinations or delusional beliefs, are persistent over time and associated with poorer prognosis [3,4]. In line with this, social support has been shown to be a protective factor in this diagnostic group, thus social deficits further compound their prognostic outcomes [5,6].

Our understanding of the social difficulties in PSz is derived primarily from studies exploring social cognition in PSz, referring to the mental operations that underpin the process of social interaction such as perception and interpretation of social cues [7]. Such studies infer social skill from performance in off-line tasks completed outside the context of social interaction. Examples include discriminating facial expressions in pictures; attributing emotional states to the protagonists in short narratives and inferring intentions in abstract problem solving contexts. PSz consistently have poor performance on such tasks [7,8], yet it is unclear how this translates to the complex and nuanced dynamics required to navigate a real-world social interaction.

### (a) Dialogue

By contrast to written language, talk in interaction is characterized by incomplete utterances, non-word fillers (*hesitation particles*: ‘uh’ ‘erm’), pauses and self-repairs such as repeated words or phrases or reformulations of the utterance in progress [9]. Such disfluencies are often taken to be symptomatic of problems with communication, particularly in terms of self-monitoring one’s own speech production [10]. However, disfluencies in an individual’s utterances are also affected by the interaction itself [11]. Self-repairs may be produced as a direct response to feedback, including non-verbal behaviour in the form of head nods, facial expressions or hand gestures, and are also themselves linked to increased gesture use in the speaker [12–15]. Much work has been done on gesture in terms of types of gesture, timing and function ([16–19] among many others). In this paper, we do not address such detailed distinctions of gesture, but take a neutral approach, for which we use automatically derived hand movement measures as a proxy (see §2(d) for details).

Additionally, dialogue involves multiple individuals who have to organize their turn-taking appropriately so that they are not all talking at once, and nor are there large gaps between turns. The classic conversational analysis (CA) account of turn-taking [20], takes speaker change to be licensed at transition relevance places (TRPs), which occur after turn constructional units (TCUs); segments of speech which are in some sense complete. However, speaker change is not obligatory at a TRP since the current speaker may continue with a new TCU. Where turn changes do occur, Sacks *et al.* [20] distinguish between cases where the current speaker selects the next speaker (for example, by directly addressing a question to someone or gazing and/or gesturing towards a specific individual as they reach a TRP) and those in which the next speaker self-selects (e.g. by answering a question that was directed to a group of people). By contrast, cue-based models [21] emphasize the types of embodied behaviours that speakers make use of to manage turn-taking behaviour, including multimodal factors such as gaze and gesture, but do not fully consider the effects of the (potential) next speaker’s behaviours. While there is some debate in the literature, there is good evidence that gaze can facilitate turn yielding in face-to-face dialogue [22], although it is not a completely reliable signal, especially in multiparty dialogue [23]. Additionally, pauses or hesitation particles are also associated with such interaction management [24], and can signal turn yielding or floor holding.

For PSz, individual tasks suggest that they have difficulty monitoring their own behaviour [25] and mismatches between speech and gesture [26]. Role-play studies also show that PSz are less effective at meshing their turns [27] and have atypical patterns of gesture [28]. Gesture performance in particular is linked to social functioning prognosis longitudinally [29]. Evidence from genuine interactions is limited, however, and contradictory, with some studies reporting that PSz use fewer self-repairs than people without a diagnosis [30], for example, and others reporting that they use more [31,32]. These studies may not be comparable owing to the contexts of the interactions; often with a therapist or interviewer who is aware of the diagnosis.

Studies using a unique corpus of triadic interactions in which half of the dialogues include a person with a diagnosis of PSz, but their interacting partners are not aware of the diagnosis (see §2), show that PSz use fewer gestures while speaking [33], and have reduced coordination between gesture and speech [34] and between gesture and repair [35]. Analysis of disfluencies on the same data [36] shows that in contrast to the evidence from therapist interactions [32], PSz use fewer self-repairs than both their interacting partners and controls (Cs) in groups without a PSz, suggesting that the context of the interaction is a key factor, rather than issues with self-monitoring *per se*. PSz and their interacting partners use fewer hesitation particles than Cs, which may be owing to reduced competition for the floor, an empirical question requiring more in depth analyses of different corpora. For unfilled pauses (defined as gaps of greater than 200 ms between utterances where the same speaker speaks before and after the gap), these are more common within the turns of people interacting with a PSz. We hypothesize that this may be because floor holding and turn yielding cues are less useful in dialogues with a PSz.

The triadic nature of the interactions in this corpus offers a unique opportunity to investigate the dynamics of turn exchange when there is competition for the position of speaker/addressee. This is not possible in dyadic interactions, and is more complex in larger multiparty interactions.

The findings derived from this corpus to date on disfluencies and gesture [33,34,36] are based on data collated at the level of participant, so cannot directly address issues around the timing or dynamics of turn-taking at the level of the intertwining utterances. The current mixed methods analysis builds on the previous analyses, investigating the dynamic nature of disfluencies, gesture and their multimodal relationship in the negotiation of turn exchange in these triadic interactions.

## 2. Methods

### (a) Data

The corpus, described elsewhere [33] consists of 40 triadic interactions. Half of these involve a PSz (six male and 14 female) and two C participants (21 male and 19 female), and the other half involve three C participants (34 male and 26 female). In each triad, people were unfamiliar to each other. In PSz interactions, C participants were unaware that they were interacting with a person who had a diagnosis of PSz. Interactions were motion captured and audio-visually recorded. All procedures were approved by a NHS Research Ethics Committee (07/H0711/90). All participants provided written informed consent. One C group and one PSz group were excluded from the analysis owing to issues with video data. A further PSz group was excluded owing to missing motion capture data.

### (b) Schizophrenia sample

Exclusion criteria included those presenting with motor side effects from anti-psychotic medication (e.g. muscle stiffness and involuntary muscle spasms) and non-fluent English speakers. Seventeen of our PSz participants were taking anti-psychotic medication (two typical; 15 atypical) and three were medication free. All PSz participants were diagnosed as having PSz and were regularly attending psychiatric outpatient appointments. The positive and negative syndrome scale for schizophrenia (PANSS) [37] assessed their positive, negative and general symptoms. PSz symptoms scores were relatively low (PANSS positive symptoms *M* = 15.8, s.d. = 6.76; PANSS negative symptoms *M* = 9.95, s.d. = 3.36; PANSS general *M* = 28.41, s.d. = 10.42). PSz participants were not displaying overt symptoms at the time of the interactions, e.g. verbally responding to auditory hallucinations.

### (c) Task

Participants were instructed to discuss a moral dilemma called the Balloon Task. This task (described in detail elsewhere, see [38]) requires participants to reach agreement on which of four passengers should be thrown out of a hot air balloon that will otherwise crash, killing all the passengers, if one is not sacrificed.

### (d) Analysis

All speech data were transcribed in ELAN [39]. The duration between utterances was extracted automatically in milliseconds (ms). Positive durations correspond to gaps and negative durations correspond to overlaps. For the turn-taking analysis presented here we only considered cases where there was a speaker change. We do not consider whether the speaker change occurs at a TRP or not—in some cases it will, in other cases it will not (for example, where there is an apparent speaker switch for a mid-turn backchannel from another speaker which will split a single turn from a speaker into two utterances, with an intervening backchannel). Of course, this has consequences for the analysis and interpretation, however, the methodology applies equally to both PSz and C groups so differences between them are still relevant and meaningful. See also [40] for discussion of related issues.

Self-repairs were annotated using strongly incremental repair detection (STIR; [41]) which automatically detects speech repairs on transcripts. STIR is trained on the Switchboard corpus [42], and has been shown to be applicable to therapeutic dialogue, with high rates of correlation to human coders in terms of self-repair rate [43].

Hand movement was automatically extracted from the raw motion capture data. In order to control for individual variation, for each participant we extracted the movement from each of the three hand/wrist markers, and calculated the mean and standard deviation (s.d.) of movement in any direction by frame in millimetres (mm). For frames with missing markers, if this was fewer than 50 frames (frame rate 60 s^−1^), we imputed the missing data using a linear trajectory, otherwise left the data as missing. Following the methodology in [33,44], to account for individual variation, for each pair of frames we calculated whether the movement between them was greater than the individual’s mean movement+1 s.d., for any of the three markers, and if so marked this as movement. The use of all three wrist and hand markers helps to mitigate the points where single markers dropped out, e.g. owing to occlusion. The hand movement data were imported to ELAN. Visual inspection of the data suggested that using an individual’s mean+1 s.d. is generally a good proxy for hand movement. However, this is not the case where this value was very low (owing to minimal or no movement, or extreme cases of marker drop out) in which case the algorithm was oversensitive to minor non-gestural movements caused by posture shifts, for example. It was also not accurate in cases where the value of the mean+1 s.d. was very high (individuals who gesture a lot), in which case the algorithm was undersensitive to genuine gestures. For this reason, we introduced a lower and upper threshold for the movement values. These were set at 2 mm per frame for the lower bound and 5 mm per frame for the upper bound. These refinements to the movement calculation result in a more reliable and sensitive index of hand movement than has been adopted in previous analyses of this corpus (e.g. [33]).

It should be borne in mind that although we believe that our automatically derived hand movement measures are a good proxy for gesture, they do not distinguish between gestural hand movement and other hand movement (e.g. scratching, fidgeting). It is also the case that the automatic hand movement annotation captures only the movement phases of a gesture—including preparation and retraction [16], and will not pick up any hold phases of gestures, which are known to be interactionally relevant (see e.g. [45]), particularly in respect to turn-taking.

Analyses were performed in SPSS 28.

## 3. Results

### (a) Turn transitions

In order to assess differences in turn-taking in the C and PSz dialogues, we compared the duration between each turn by group, participant type and who speaks next.

**Figure 1.**
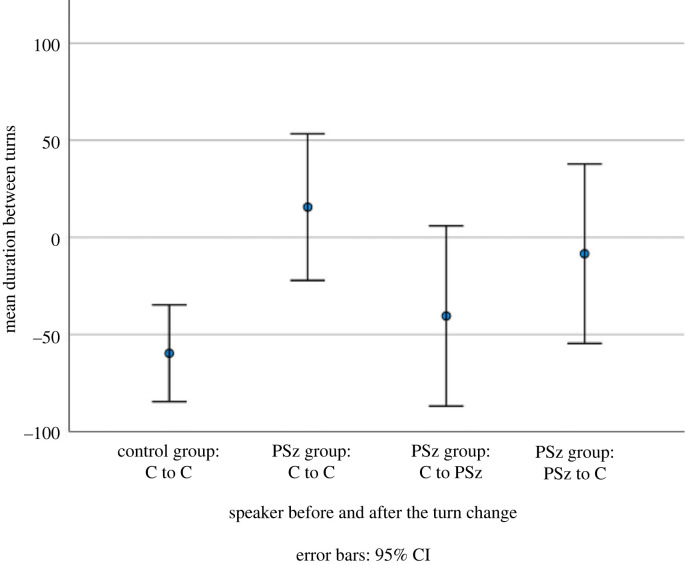
Turn change duration by speakers before and after the turn change.

As seen in table 1, both the PSz and C groups have turn changes which are on average below zero (i.e. in overlap). One-way ANOVAs show that there is a significant difference between the groups with turn exchanges in the C groups occurring faster than those in the PSz group (*f*_1,7068_ = 8.513, *p* = 0.004). However, drilling down in the PSz groups suggests that this is not the complete picture. While there is no significant difference in the gap following a turn by the PSz and Cs in the PSz groups, this masks the difference which emerges when we look not only at the identity of the person who speaks the turn *prior* to the turn change but also the turn *following* the turn change, as shown in figure 1 (*f*_3,7066_ = 3.880, *p* = 0.009). Post hoc tests with a Bonferonni correction show no significant differences between groups except for between C to C in the control and PSz groups (*p* = 0.008, 95% confidence interval (CI) −137.31 to −13.29). This means that turn exchanges between the two Cs in the PSz group have a longer gap than turn exchanges between Cs in the C group.

**Table 1.** Turn change duration by group, participant type and speakers.

		mean	s.d.	*n*
group	control group	−59.66	780.56	3776
	PSz group total	−7.18	723.49	3294
participant	PSz in PSz group	−8.38	720.55	937
type	C in PSz group	−6.70	724.81	2357
next	C to C in PSz group	15.64	724.73	1418
speaker	C to PSz in PSz group	−40.44	724.01	939

These results, particularly in conjunction with the less fine-grained results presented in [36], provide evidence that in the dialogues with a person with a diagnosis of PSz, there are differences in the timing of turn transitions. We hypothesize that this could indicate that the cues for turn ending (or specific next speaker selection) are less clear in the PSz interactions, i.e. they may be missing or ambiguous. It could also be the case that what the PSz has said may be more difficult to formulate an appropriate response to. It is particularly striking that the turn exchanges most affected by the presence of a PSz are those between their C interlocutors. We interpret this as suggesting that there are specific points (such as TRPs; [20]) at which the Cs in the PSz groups are expecting (or encouraging) the PSz to take the turn but the PSz is not doing so, with the result that after a pause the other C steps in. However, more detailed analyses of whether the gaps are consistently occurring at TRPs is required to validate this interpretation. This may also suggest that PSz are less responsive to turn-taking cues or more reluctant to select as next speaker, but again more targeted analyses are required to ascertain if this is the case.

### (b) Qualitative analysis

Alongside previous results on disfluencies, the above quantitative analysis suggests that in PSz interactions there are bigger gaps between turns between the two Cs in interactions including a PSz. But what does this mean in practice? We now turn to some examples from our data. Note that our examples are for illustrative purposes only. We do not claim that our discussion of them is exhaustive and nor do we describe the examples in the level of detail of CA.

#### (i) Passing up the opportunity to take the floor

**Figure 2.**
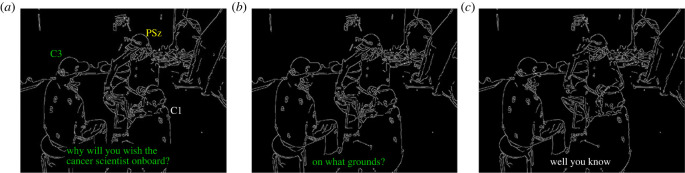
PSz non-response to a question.

The clearest case of the pattern of pauses, which we hypothesize to be more common in the PSz interactions, is that where the PSz is passing up an apparent opportunity to take the floor, which is subsequently taken by the other C participant, after a gap. An example of this can be seen in figure 2 and table 2. In this example, C3 produces the first part of a question-answer adjacency pair [46,47] in line 3, table 2. This creates the expectation for the second pair part—namely an answer. As can be seen in figure 2*a*, as C3 is asking the question, both the PSz and C1 are looking at C3 as he speaks. C3’s gaze and postural orientation is towards the PSz, which is taken as an indication—at least by C1 who does not immediately proffer an answer to the question—as C3 selecting PSz as the next speaker [23]. In line 4 (figure 2*b*), following a short gap in which none of the participants alters their posture or shifts their gaze, and no answer is forthcoming from PSz, C3 further specifies his initial question, increasing the expectation of a response. After approximately a second of the 2.39 s gap in line 5, rather than providing an answer, which both C3 and C1 seem to expect, PSz leans back slightly and shifts his gaze towards C1, at which point she takes the floor and provides the second part of the original adjacency pair. The 2.39 s gap in line 5 is over twice as long as the 1 s ‘standard maximum’ silence in dialogue proposed by Jefferson [48], and such a long silence may go beyond a gap to become a lapse in the conversation [49]. In this case, it is only after the standard maximum silence duration that the PSz shifts his posture and gaze and C1 appears to take this as an indication that her expectation that he would answer the question posed by C3 is incorrect, leading to her self-selection as next speaker.^1^

**Table 2.** PSz passing up the opportunity to take the floor by not responding to a direct question.

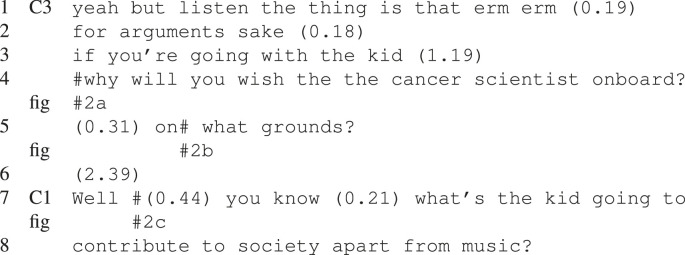

#### (ii) Explicit turn exchange cues

**Figure 3.**
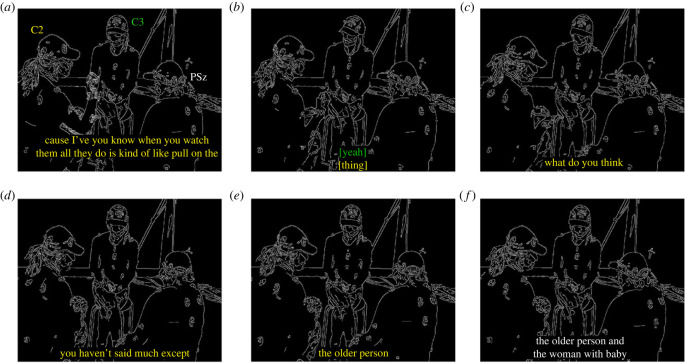
Increasingly explicit turn cues.

The example sequence in figure 3 and table 3, shows one of the strategies adopted by the Cs in dialogue with a PSz. At the beginning of this example, in (figure 3*a*) the speaker, C2, is actively orienting her body, gaze, head and gestures towards the PSz, with C3, also orienting towards the PSz. The PSz is unresponsive and as C2 begins to articulate the final word of the utterance in line 1–2 (‘thing’) she turns her gaze towards C3, who simultaneously turns her gaze towards C2 and provides an acknowledgement in overlap (figure 3*b*), line 3, as well as a non-verbal nodding response. This creates an environment in which the agreement or disagreement of the PSz becomes relevant—even without C2’s bodily orientation towards her, which the PSz does not take up in the following 1.81 s long gap. Interestingly, even before C3’s agreement, C2’s utterance in line 1–2 (which is oriented towards the PSz) seems to be looking to elicit agreement from the PSz, as it builds on a recent previous utterance from her; less than 10 s previously the PSz stated ‘I don’t reckon it’s that hard to fly a balloon’.

**Table 3.** Increasingly explicit turn exchange cues from Cs towards PSz.

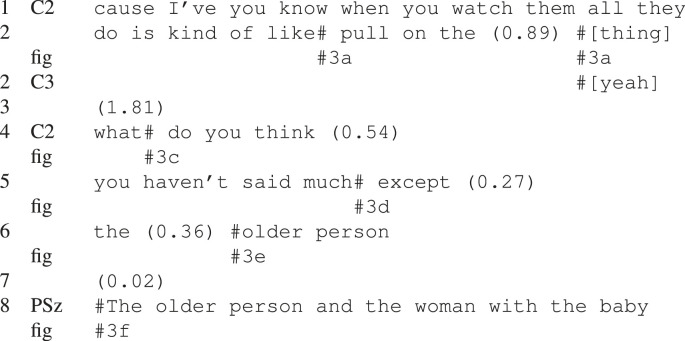

Towards the end of the long gap, C2 turns her gaze back towards the PSz, followed by asking her a direct question (figure 3*c*), line 4. As in the previous example, this first pair part sets up the expectation for the second part of the adjacency pair. This means that the PSz no longer simply has the opportunity to take a turn, but there is a stronger—socially normative—obligation for her to do so. At the start of the question in line 4, C3 is still gazing at C2, but she turns her gaze towards the PSz over the (1 s) duration of the question, showing that she also orients to the expectation of a response from the PSz [50].

Following a 540 ms gap where the expected response is not forthcoming, C2 provides, in line 5, a reason for the question, which demonstrates that she expected the PSz to take a turn. The PSz once again avoids the now more explicit turn-taking cue by actively looking away, precisely as C2 begins the utterance in line 5 ‘you haven’t …’ (figure 3*d,e*), turning her gaze back to C2 just after C2 utters ‘older’ in line 6 before finally taking the floor in (figure 3*f*), line 8. As can be seen in this example, the Cs are doing a lot of interactive work to include the PSz in the interaction.

Taken together, the quantitative results and these examples suggest that there are cases in the PSz interactions where there are differences in turn-taking patterns. This may be because the PSz lacks awareness of the normative turn-taking expectations, or because they are actively avoiding cues directed towards them.

We now turn to the relationship between gesture and repair in dialogues with a PSz.

### (c) Hand movement and repair

As discussed, previous work shows that PSz have decreased gesture use while speaking [33] and use fewer self-repairs than Cs [36], even when normalized for the fact that PSz speak less than their interlocutors. One possible explanation for the reduction in gesture in PSz is precisely the link with repair, since people typically increase their gesture use in problematic turns, as indexed by rates of repair [51].

**Figure 4.**
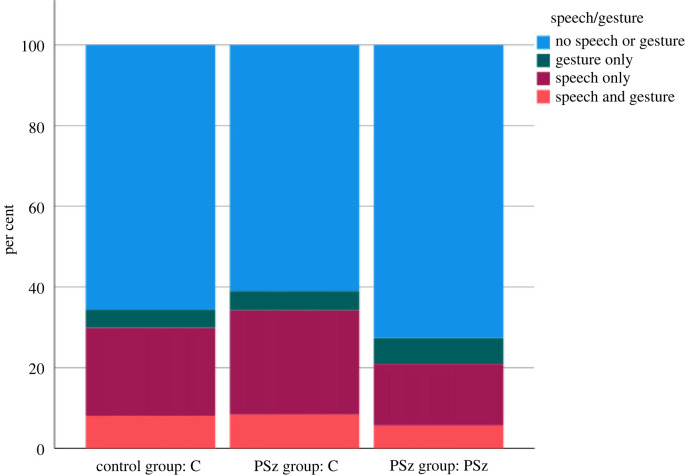
Gesture and/or speech by number of frames. (Online version in colour.)

**Figure 5.**
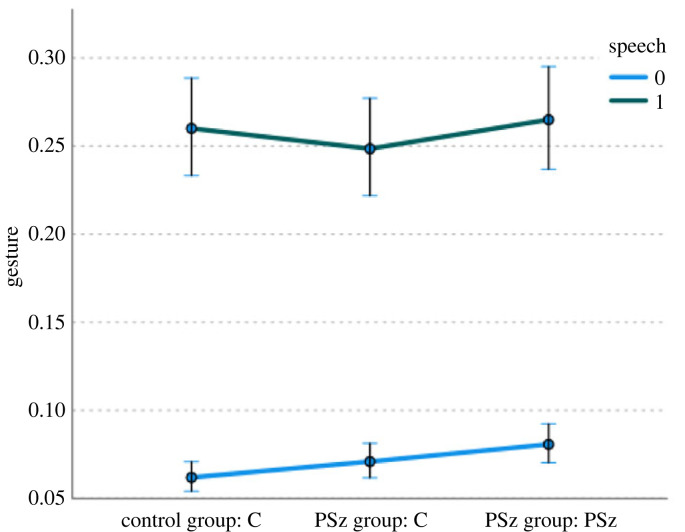
Gesture by speech and group/condition. (Online version in colour.)

Firstly, we should note that, in contrast to previously reported results based on participant level values and a less sensitive measure of gesture [33,35], we did not find that PSz gestured less as speakers. When we look at hand movement on a frame-by-frame level (see figure 4), we find that PSz gesture *more* while not speaking, while the Cs in the PSz group gesture proportionally *less* while speaking, as shown in figure 5. A generalized linear mixed model (GLMM; with a binomial probability distribution and logit link function) by frame with presence or absence of hand movement as the dependent variable, presence or absence of speech and group/condition as fixed factors, and triad as random variable^2^ showed a significant main effect of speaking (*f*_1,1062168_ = 44607.14, *p* > 0.001), such that hand movement is much more likely when someone is speaking, a significant main effect of group/condition (*f*_2,1062168_ = 66.02, *p* > 0.001), such that PSz produce more gesture than their C interlocutors, and a significant interaction effect of group and condition by speaking (*f*_2,1062168_ = 106.86, *p* > 0.001), with significant differences in the non-speaking condition between the PSz in the PSz group and both Cs in the PSz group (*p* < 0.01) and Cs in the C group (*p* = 0.016) and in the speaking condition between the PSz and Cs in the PSz group (*p* < 0.001). This means that PSz produce more gestures than both C groups when not speaking, and their interacting partner Cs produce fewer gestures than the PSz when speaking.

**Figure 6.**
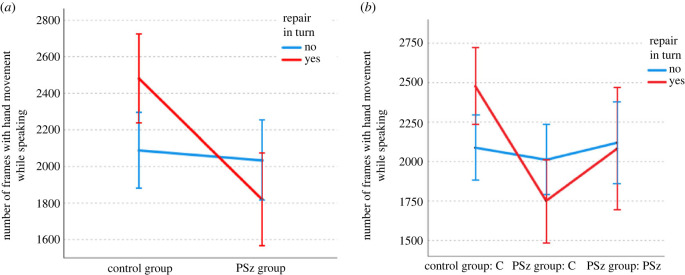
Gesture by repair and group/condition and next speaker. (Online version in colour.)

Turning to the relationship between gesture and repair (looking only at the hand movements which co-occur with speech), we can see that the presence of repair in a turn is a good predictor of amount of hand movement, but only in the C groups, as shown in figure 6. A GLMM (using a normal distribution and identity link function) with number of speaking frames containing hand movement as the dependent variable, group type and presence or absence of repair and number of words as independent variables and triad, age and gender as random effects showed a main effect of group type (such that C groups contain more hand movements: *f*_1,7103_ = 5.23, *p* = 0.022), a main effect of number of words (such that longer turns are more likely to have more hand movements: *f*_1,7103_ = 4992.50, *p* < 0.001) and an interaction between group type and repair (*f*_1,7103_ = 28.40, *p* < 0.001, such that there was a difference between the groups in the repair condition, but no significant difference in turns without repair: *t* = 3.68, *p* < 0.001).^3^ This means that there is less hand movement in turns with repair in the PSz group than in the C group, and demonstrates that the main effect of group is driven by the differences in turns which contain repair. There was no main effect of repair (*f*_1,7103_ = 2.08, *p* = 0.150).

Interestingly, when we drill down by participant type, we see that the effect is more pronounced in the Cs in the PSz group (figure 6*b*), with amount of hand movement of a PSz unaffected by the presence or absence or repair. A GLMM with the same settings and factors except with group/condition instead of group showed the same pattern of main and interaction effects.^4^ Post hoc tests show that in the turns with repair, Cs in the C group use more gesture than Cs in the PSz group (*t* = 3.95, *p* < 0.001), and that Cs in the C group use more gesture in turns with repair (*t* = 4.756, *p* < 0.001) than in turns without repair, in line with previous findings. By contrast, Cs in the PSz group show the opposite pattern, producing less gesture in turns containing repair (*t* = −2.66, *p* = 0.008). We will return to the possible reasons for this in the discussion.

**Figure 7.**
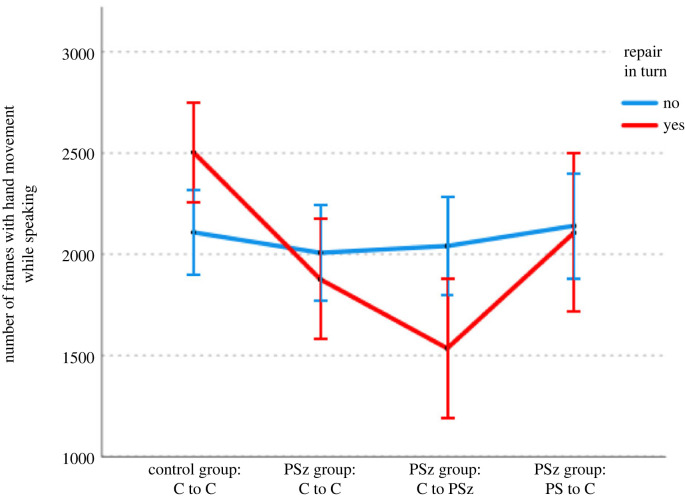
Gesture by repair and group/condition. (Online version in colour.)

Further, this difference is sensitive to the turn-taking dynamics discussed in §3(a). As shown in figure 7, when we also consider who is the next speaker,^5^ we see that the unexpected effect of Cs producing less gesture in turns containing a repair only holds when the PSz is the next speaker (*t* = −3.289, *p* = 0.001).

## 4. Discussion

This mixed methods analysis provides an in-depth investigation of turn exchange behaviour in triadic interactions involving a person with a diagnosis of PSz. The findings demonstrate that PSz’ turn-taking behaviour deviates from expectations, in often subtle ways, resulting in an adaptation by others. Interactions involving PSz showed reduced competition for the floor, and a lack of clarity about who should take the floor. PSz’ use of floor change cues deviates from C participants’ expectations; missing or avoiding subtle turn exchange cues and failing to provide such cues during their own turns. This difference in response time is also consistent with work which suggests that competition for the floor in multiparty interactions usually reduces turn exchange times [52]—as is the case in the C groups, but not in the PSz groups, where competition seems to be reduced.

We hypothesize that the differences seen in the turn-taking behaviours may be because the PSz lacks awareness of the normative turn-taking expectations, or because they are actively avoiding cues directed towards them: our analysis here, based on an automatic detection of silences between turns, does not distinguish between gaps which occur at a TRP and those that do not, which may be a factor here (though note that our broad observations about differences between the groups hold despite this lack of nuance). Future work will address these questions using both human annotation and recently developed automatic methods to detect TRPs [53], and will also consider the content and type of turns [38,54], since our qualitative analysis suggests that adjacency pairs may be used in the PSz dialogues to make turn-taking cues more explicit.

Furthermore, the multimodal coordination between gesture use and self-repair that persists in C interactions, is not present in interactions involving a person with PSz. PSz’ use of gesture was not related to their use of self-repair, while Cs interacting with a PSz displayed *fewer* gestures in utterances that included self-repair. Overall, the presence of a person with a diagnosis of PSz in a social interaction changes the behaviour of those they are interacting with, despite their diagnosis being undisclosed.

Our findings align with those of previous studies of communication in PSz derived from role-play or task-based methodologies, specifically, PSz have difficulty meshing their turns [27], have deficits in their use of gesture [28] and a mismatch between gesture and speech [26]. This study demonstrates that these difficulties persist in naturalistic social interaction and have a significant influence on others’ communication. Furthermore, research from the field of social cognition suggests that PSz have difficulty interpreting social cues in pen and paper assessments [7]. Although our analysis does not investigate PSz’s ability to interpret social cues, it does suggest that they may fail to use such cues when offered in conversation.

Our previous analysis of this corpus was at the level of participant and the index of gesture used speed of movement [33]. When manually inspecting the gesture categorization alongside the video footage this method was found to under and/or over categorize hand movements as *‘gesture’*. As such, in the current analysis, we employed a more sensitive measure of hand gesture based on the raw motion capture data of three motion capture markers, rather than one, alongside manual inspection of the data. This analytic approach revealed that, compared to Cs in either group PSz displayed more hand movements categorized as ‘gesture’ when they were not speaking. Observational annotation of these movements would be required to identify their nature, however, one working hypothesis is that these movements are indicative of displacement behaviours, which are self-directed behaviours, found to be correlated with states of heightened arousal, or anxiety (e.g. [55]). This raises potential questions about the validity of our methodology, when interpreted as ‘gesture’, as discussed in §2. However, we believe that our methods bring advantages in terms of the scale of data that can be analysed without time-consuming human annotation effort. We also believe that the insights into the shifting dynamics through an interaction brought out by our automatic hand movement detection can be complementary to more traditional gesture annotation and qualitative methods.

In the current analyses, Cs interacting with a PSz produced *fewer* hand gestures when speaking. Taken alongside the lack of competition for the floor, this pattern may suggest a reduction in the need to employ floor-holding techniques such as hand gesture. Although this may be the case, drilling further into the turn exchange dynamics we also identified a disruption in the relationship between gesture and speech in both PSz and their C partners, compared to the C group, where we saw the expected relationship between repair and increased gesture use (in line with [14,51]).

The most prominent finding is seen in turn exchanges where Cs pass their turn to the PSz; here Cs employed significantly fewer gestures when they have verbal difficulty, as indexed by the use of self-repair. In interactions with a PSz, the relationship between speech repair and gesture is disrupted, not only for PSz but for the interaction as a whole. This suggests that when passing a turn to a PSz, Cs prioritize use of one modality rather than coordinating modalities.

This is puzzling. We hypothesize that the use of gesture in turns which contain self-repair is a normally productive strategy, signalling either the presence of a potential problem or the attempt to resolve it. In interactions with a PSz, where turn exchange may have greater ambiguity, this strategy does not seem to be employed. This might suggest that its usefulness is overridden by other considerations in the interaction. Although the current analyses do not identify the reason for this, we present a number of possible explanations. For example, if the potential misunderstanding involves the PSz, which may be more likely in cases where they take the next turn, their diminished responsiveness, potentially even a lack of shared gaze (see the example in figure 3), may mean gestures are less useful in this context, with more explicit verbal requests being favoured as an alternative to engage the PSz. One way to potentially unpick this in PSz interactions where turn exchange is ambiguous would be to analyse turns, not by next speaker, but by the identity of the direct addressee. This would enable us to see if the reduction in coordination between repair and gesture remains when the turn exchange is more predictable and the addressee is another C, rather than the PSz. Other possible factors which could be investigated in future work are whether the types of repairs are the same (for example, there may be less need for gestural support for articulation repairs) or whether the Cs in interaction with a PSz change their behaviour during the course of dialogue. Another possible direction for future analysis which can also be conducted on the motion capture data, is identifying the types of movements. In [15], they found that maximum hand heights for speakers were higher during disfluencies compared to other moments in interaction, suggesting that there are particular types of gestures associated with repair. If these gestures are interactive in nature (analogously to the verbal hesitation particles which act as a floor-holding device), then this might also be a factor in their reduced use when passing the floor to the PSz, owing to the already discussed lack of competition for the floor.

The current analysis did not investigate the reasons for the behaviours observed, in either turn-taking or gesture and repair, nor do we suggest that any behavioural pattern is superior to another. This study merely presents an account of how communication in interactions involving PSz differs from those involving Cs. We do not know if the nature of the task, or the fact that it took place in a laboratory environment contributed to a level of anxiety in PSz or how this may have contributed to PSz’ behaviour. Future comparative studies in different contexts, using different conversational topics could explore these questions.

## 5. Conclusion

These investigations demonstrate the complex interconnectedness of participants in an interaction and the necessity to analyse them as a dynamic unit [56,57]. As well as offering some insights into how the presence of a PSz affects an interaction as a whole, they also demonstrate the flexibility of our mechanisms for interaction. As the Cs conversing with a person diagnosed with PSz show, strategies can be—and are—adjusted on the fly to account for deviations in the expected interactive behaviours from one’s interlocutors. As usual, there is much work still to be done.

## Data Availability

This article has no additional data.

## Endnotes

1 We thank our anonymous reviewer for this point.

2 Models including frame number as an additional effect did not alter the main pattern of results of account for any more of the variance in the data so we report models without these here.

3 Post hoc tests were carried out using pairwise comparison and the Bonferroni correction.

4 Main effect of number of words: *f*_1,7101_ = 4991.74, *p* < 0.001; group/condition *f*_2,7101_ = 4.39, *p* = 0.012 and interaction effect group/condition by repair *f*_1,7101_ = 14.48, *p* < 0.001.

5 Once again, the pattern of significant effects is the same: number of words: *f*_1,7061_ = 5085.76, *p* < 0.001; group/condition/next speaker *f*_3,7061_ = 4.13, *p* = 0.006 and interaction effect group/condition/next speaker by repair *f*_3,7061_ = 11.52, *p* < 0.001.
